# *Anopheles coluzzii* larval habitat and insecticide resistance in the island area of Manoka, Cameroon

**DOI:** 10.1186/s12879-016-1542-y

**Published:** 2016-05-20

**Authors:** Josiane Etang, Arthur Mbida Mbida, Patrick Ntonga Akono, Jerome Binyang, Carole Else Eboumbou Moukoko, Leopold Gustave Lehman, Parfait Awono-Ambene, Abdou Talipouo, Wolfgang Ekoko Eyisab, Darus Tagne, Romeo Tchoffo, Lucien Manga, Remy Mimpfoundi

**Affiliations:** Institut de Recherche de Yaoundé (IRY), Organisation de Coordination pour la lutte contre les Endémies en Afrique Centrale (OCEAC), B.P. 288, Yaoundé, Cameroun; Biological Sciences Unit, Faculty of Medicine and Pharmaceutical Sciences, University of Douala, P.O.Box 2701, Douala, Cameroon; Laboratory of Animal Biology and Physiology, Faculty of Science, University of Douala, P.O.Box 24157, Douala, Cameroon; Pôle d’Excellence en Epidémiologie du Paludisme, Service d’Epidémiologie et de Santé Publique, Centre Pasteur du Cameroun, B.P. 1274, Yaoundé, Cameroun; World Health Organization, Regional office for Africa, P.O.Box 6, Cité Djoué, Brazzaville Congo; Laboratory of General Biology, University of Yaounde I, P.O.Box 812, Yaounde, Cameroon

**Keywords:** Ecology, Malaria, Mosquitoes, Insecticide resistance, Cameroon, Island, Africa, Integrated vector management

## Abstract

**Background:**

The effectiveness of Long-Lasting Insecticidal Nets and Indoor Residual Spraying in malaria vector control is threatened by vector resistance to insecticides. Knowledge of mosquito habitats and patterns of insecticide resistance would facilitate the development of appropriate vector control strategies. Therefore, we investigated *An. coluzzii* larval habitats and resistance to insecticides in the Manoka rural island area compared with the Youpwe suburban inland area, in Douala VI and II districts respectively.

**Methods:**

Anopheline larvae and pupae were collected from open water bodies in December 2013 and April 2014 and reared until adult emergence. Two to four day old emerging females were morphologically identified as belonging to the *An. gambiae* complex and used for WHO susceptibility tests with 4 % DDT, 0.75 % permethrin, and 0.05 % deltamethrin, with or without piperonyl butoxide (PBO) synergist. Control and surviving specimens were identified down to the species using a PCR-RFLP method. Survivors were genotyped for *kdr* L1014 mutations using Hot Oligonucleotide Ligation Assay.

**Results:**

In both study sites, ponds, residual puddles, boats, and drains were identified as the major *An. gambiae s.l.* larval habitats. A total of 1397 females*,* including 784 specimens from Manoka and 613 from Youpwe, were used for resistance testing. The two mosquito populations displayed resistance to DDT, permethrin and deltamethrin, with variable mortality rates from 1 % to 90 %. The knock-down times were also significantly increased (at least 2.8 fold). Pre-exposure of mosquitoes to PBO did not impact on their mortality to DDT, conversely the mortality rates to permethrin and deltamethrin were significantly increased (7.56 ≤ X^2^ ≤ 48.63, df = 1, *p* < 0.01), suggesting involvement of P450 oxidases in pyrethroid resistance. A subsample of 400 *An. gambiae s.l.* specimens including 280 control and 120 survivors from bioassays were all found to be *An. coluzzii* species. Only the *kdr* 1014 F mutation was found in survivors, with 88.5 % (*N* = 76) and 75 % (*N* = 44) frequencies in Youpwe and Manoka respectively.

**Conclusion:**

This is the first report of *An. coluzzii* resistance to insecticides in an insular area in Cameroon. Since permanent larval habitats have been identified, larval source management strategies may be trialed in this area as complementary vector control interventions.

## Background

Despite several decades of control efforts, malaria remains a major public health problem in most tropical and subtropical regions of the world, especially in Africa [1]. The uncontrolled population growth in many areas has led to extensive deforestation, irrigation, and unplanned urbanization. These high populations and associated environmental modifications create ecological conditions favouring the proliferation of arthropod vectors, including malaria-transmitting mosquitoes [2]. Vector control, mainly based on the extensive use of Long-Lasting Insecticidal Nets (LLINs) and Indoor Residual Spraying (IRS), is a corner stone in current malaria control and elimination strategies [1]. With adequate knowledge of larval habitats, these two weapons can be complemented effectively with larval source management under a specific set of environmental conditions [3].

In Cameroon, malaria annually accounts for 35–40 % of deaths in health facilities, 40–45 % of out-patient consultations, and 30 % of hospitalisations [4]. It is also responsible for 26 % of job and school absenteeism and over 40 % of domestic health expenses [5]. Malaria infection is essentially due to *Plasmodium falciparum*, followed by *P. ovale* and *P. malariae* [6]*.* Seven anopheline species play major roles in *Plasmodium* parasite transmission, among which are *Anopheles (An.) arabiensis*, *An. gambiae* and *An. coluzzii*, three sibling species of the *An. gambiae* complex [7]. *Anopheles arabiensis* is mostly found in the Northern savannah Regions, while *An. gambiae s.s.* and *An. coluzzii* are widespread throughout Cameroon [8]. Although *An. gambiae s.s.* and *An. coluzzii* share the same resources such as vertebrate hosts or freshwater habitats, they have been shown to diverge in some of their biological and ecological requirements [9–11]. In the dry savannahs of West Africa, *An. gambiae s.s.* preferentially breeds in temporary aquatic habitats and is found during the rainy season only, whereas *An. coluzzii* is present all year round, breeding in man-made permanent aquatic habitats [10, 11]. Species are sharply segregated along a gradient ranging from “permanent-anthropic” habitats, exploited by *An. coluzzii* to “temporary natural” habitats where *An. gambiae s.s.* and *An. arabiensis* are found [12]. Consequently, larval ecology of this species complex appears clearly linked to habitat hydro-periodicity and to the ecological communities they support in relation to anthropogenic activities. In Cameroon, little is known about habitat segregation between *An. gambiae* s.s. and *An. coluzzii* in Island areas.

In the framework of malaria elimination, large-scale vector control interventions based on wide use of LLINs are being implemented by the National Malaria Control Programme (NMCP) in Cameroon [13]. Indeed, a nationwide free distribution of eight and a half million LLINs was carried out in 2011 by the NMCP, and a second nationwide distribution of twelve million LLINs is ongoing since 2015. However, several studies have reported insecticide resistance in *An. gambiae s.s., An. arabiensis* and *An. coluzzii* in the Northern, Central, Southern, Eastern, Western and Coastal Regions of Cameroon [14–16]. The resistance to DDT and pyrethroid insecticides reported in species of the *An. gambiae* complex has been conferred by elevated activity of esterases, glutathione s-transferases, P450 oxidases, and *kdr* L1014F or L1014S mutations, with some populations displaying multiple resistance mechanisms [17–19]. The ongoing selection and spread of insecticide resistance in Cameroon is seen as a significant threat impeding progress towards malaria elimination in the country. To develop a comprehensive resistance management plan, it is essential to understand the spatio-temporal distribution of insecticide resistance across the country, and to continuously monitor resistance emergence in representative ecological areas. These data would be used to guide the choice of insecticides for effective malaria vector control. Until recently, the malaria entomological profile in the Manoka island was unknown, despite the potential of this setting as an experimental site for malaria elimination trials, due to its small size, insular position and manageable connections with the mainland. The current study was aimed at filling the knowledge gap on malaria vector larval habitats and insecticide resistance in the Manoka island and Youpwe crossroad quarter in mainland Douala. We report here the occurrence of permanent active larval habitats and multiple insecticide resistance mechanisms in the two *An. coluzzii* populations, including at least the *kdr* 1014 F mutation and P450 oxidase-based mechanisms.

## Methods

### Study sites

**Fig. 1:**
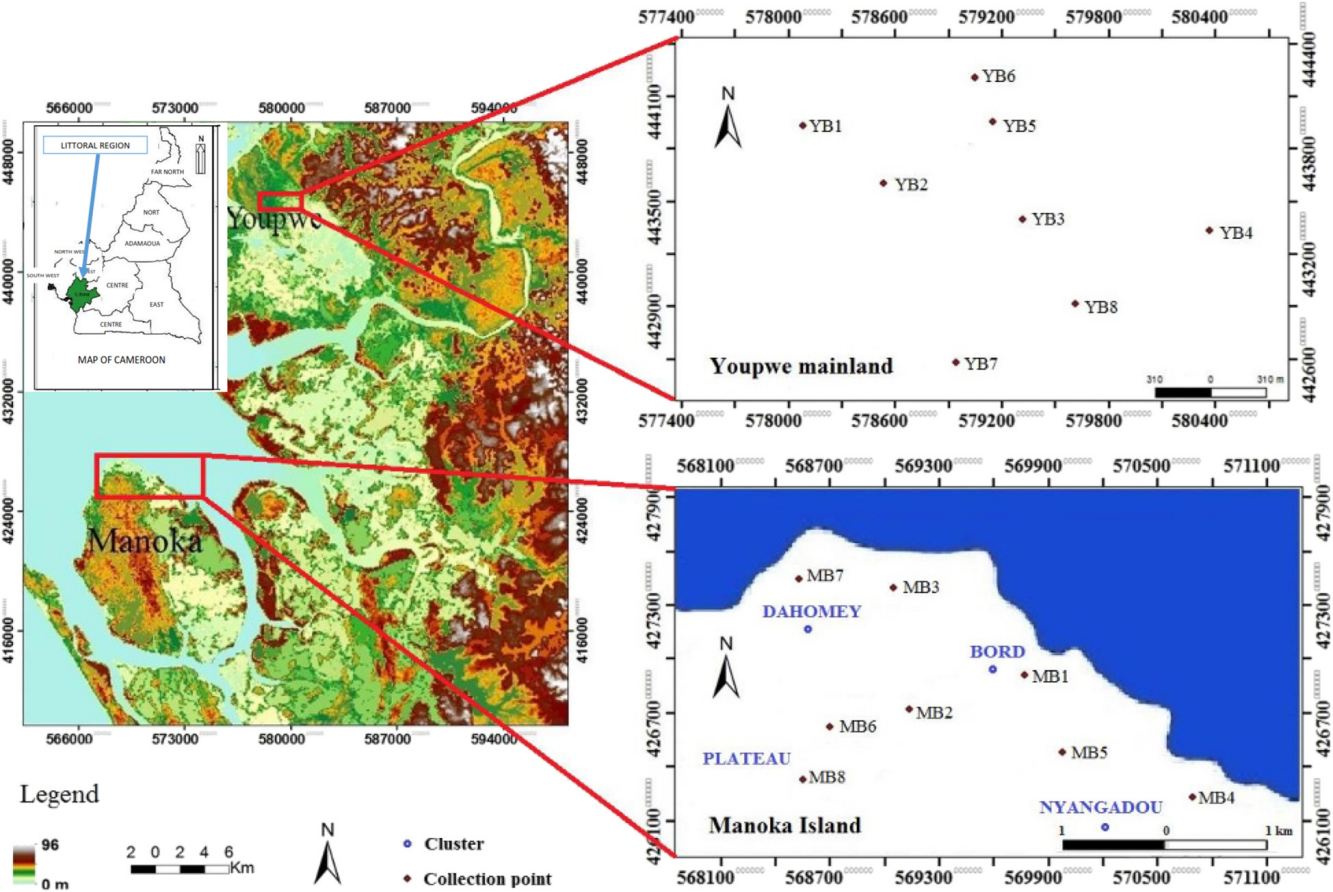
Map of the Wouri River estuary [20] showing the location of larval inspection sites. MBx: Manoka breeding site number; YBx: Youpwe breeding site number

The study was carried out in the Manoka island area (03°47' N; 09°39' E) and the Youpwe quarter (04°00' N; 09°42' E) in Douala, the economic capital city of Cameroon. Both study sites are located across the Wouri River estuary (Fig. 1) [20]. The area is characterized by a typical equatorial climate with abundant precipitation (3600–10000 mm annual rainfall) [21] and two rainy seasons (March-June, September-November) alternating with two dry seasons (December-February, July-August). The mean annual range of temperature is 27–29 °C [22], and the hydrographic network is dominated by the Wouri and Dibamba Rivers [23]. The landscape is characterized by rainforest on the banks of the Wouri River with dense mangroves, followed by degraded secondary forest on the edge of inhabited areas.

**Fig. 2:**
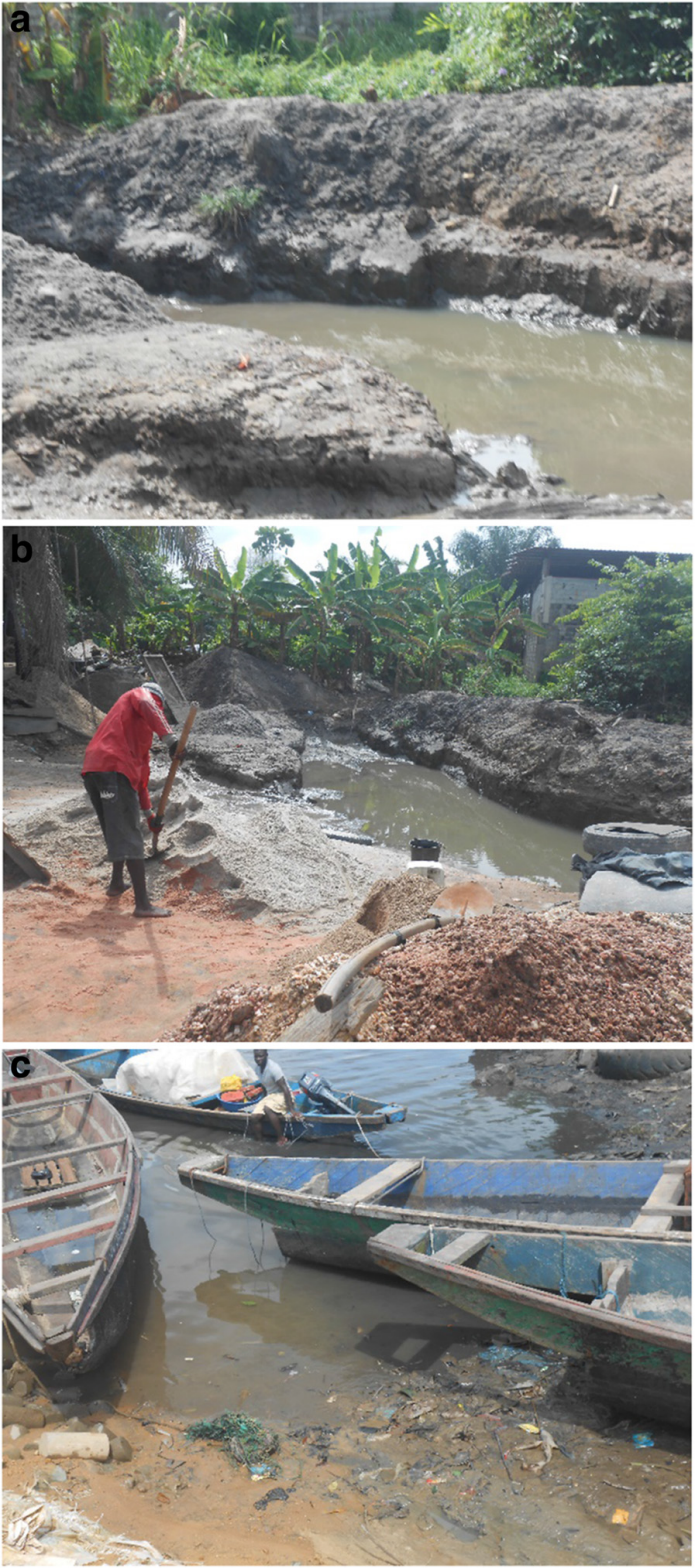
Open water bodies in Youpwe and Manoka. **a** Large drain channels in Youpwe; **b**: Sand mining pits in Youpwe; **c**: Puddles and canoes in Manoka

Youpwe (1.3 km^2^, 0.45 km^2^ inhabited) is a densely populated quarter located in the Douala II district. The local human population has been growing rapidly from 250 inhabitants in 1978–3200 inhabitants in 2005; it is anticipated to exceed 5000 inhabitants in 2045 [24]. The abundance of resources in this marine and coastal area (cheap land, timber, fish and sand) attracts people from abroad. As a result, there is an unsustainable exploitation of resources, a regression of the vegetation cover in favor of human habitations and thorough modifications of the environment (Fig. 2). Youpwe serves as a crossroad for trade activities between Douala town and the Manoka island. It is one of the fishing settlements on the outskirts of Douala that dot the coast of West Africa. A dock allows the flow of human populations and goods from Youpwe in the direction of Manoka and vice versa. The natural environment is a suburban type, with swampy soils. Besides the traditional activities like fishing, cutting and sale of mangrove wood (≈600 000 m^3^ of wood per year) and extraction of sand (≈24 000 m^3^ sand extracted per year) are increasing [24].

The Manoka island (40 000 inhabitants, 365 km^2^) was discovered in 1929; it is the largest island in Cameroon. Located off the Youpwe small fishing port, it is the sixth district of the Urban Community of Douala (Douala VI) located at ≈ 20 km and 35 min by boat from Youpwe. This island includes 10 camps (Bord, Plateau, Dahomey, Nyangadou, Epaka I & II, Buea I, Sandje, Number One Creek and Number Two Creek), with many houses built on stilts. Fishing is the main activity of local communities, followed by trade, hunting and subsistence agriculture. During the 1950s, wood and oil were exported from this island.

In both study sites, the population is composed of native Cameroonians (Bakoko and Malimba) and people from abroad (Nigerians, Ghanaians, Malians, etc.). Mosquito densities are particularly high due to proliferation of breeding sites as a result of environmental modifications by humans (Fig. 2) [24, 25]. People frequently use coils, mats and insecticide impregnated nets for protection against mosquito bites (Mbida, personal communication). Poor sanitation among human populations exposes them to severe health problems, including malaria (62.85 %), gastro enteritis (22.85 %) (amoebiasis, ascariasis, bacterial diarrhea), typhoid fever (11.42 %), urogenital infections (2.85 %) and bronchopneumonia [24].

### Mosquito sampling and morphological identification

Two field visits were conducted: one during the dry season in December 2013 and the other during the rainy season in April 2014. In each study site, all open water bodies were inspected across a total area of ≈ 1 km^2^ in Youpwe and ≈ 1.5 km^2^ in Manoka. Mosquito larval habitat types were categorized into stream, small and large drain channels, ponds, boats, swamps, rock pools, puddles, burrow pits, rain pools, stream-bed pools, wet meadows, artificial holes, concrete holes, and artificial containers. They were subdivided into permanent, semi-permanent and temporary pools, referring to any body of water that was likely to remain inundated for at least one month, between one month and one week, and less than one week respectively according to Stein et al. [26]. Location and elevation of each permanent or semi-permanent and active habitat type were recorded using a hand-held global positioning system (Garmin Inc.). Anopheline larvae and pupae samples were collected by dipping from active breeding sites [27]; samples were pooled per study site and brought to a local insectary, then reared in spring water until adult emergence. Adult anophelines were then morphologically identified as belonging to the *An. gambiae* complex by means of reference keys [28, 29] and used for susceptibility tests.

### Insecticide susceptibility testing

Susceptibility of adult mosquitoes to insecticides was assessed using WHO test kits and standard procedures [30]. Test kits including impregnated papers, test tubes and accessories were purchased from the WHO reference center at the Vector Control Research Unit, University Sains Malaysia. Filter paper sheets (12 x15 cm, Whatman N°1) were impregnated with discriminating dosages of insecticides (4 % DDT (organochlorine), 0.75 % permethrin or 0.05 % deltamethrin (pyrethroids), mixed with acetone and silicon oil. Other batches of filter paper sheets were impregnated with acetone + silicon solution for use as control. Acetone acted as the solvent and silicon oil as a carrier. The purchased impregnated papers were stored at 4 °C until the date of the test.

For each test, four batches of 20–25 non blood fed, two to four day old females were exposed to a discriminating dosage of insecticide and two batches of 20–25 non blood fed mosquitoes were used as a control. During exposure to insecticides, the number of mosquitoes knocked-down was recorded at 5-min intervals. After 1 h exposure to insecticide-impregnated papers or control papers, mosquitoes were transferred to holding tubes and provided with cotton pads soaked with 10 % sugar solution. The mortality rates were determined 24 h post exposure. Tests were performed under ambient room temperature (25–28 °C) and relative humidity of 70–80 %. Additional tests were carried out with four batches of 20–25 non blood fed mosquitoes exposed for 1 h to 4 % piperonyl butoxide (PBO) synergist, prior to exposure to each insecticide.

Susceptibility tests were concomitantly performed with the Kisumu susceptible reference strain of *An. gambiae s.s.* maintained in the Organisation de Coordination pour la lutte contre les Endémies en Afrique Centrale (OCEAC) (Yaoundé, Cameroon) insectaries. For each insecticide, four batches of 20–25 non blood fed mosquitoes were tested and two batches of 20–25 specimens as control. Tested samples were stored in Eppendorf tubes (one specimen/tube) with desiccant and kept at −20 °C for molecular analyses.

Resistance status was evaluated according to the WHO criteria [30], which classify mortality rates less than 90 % as indicative of resistance while those greater than 98 % as indicative of susceptibility. Mortality rates between 90–98 % suggest the possibility of resistance that needs to be verified. The knock-down times are considered an additional indicator of resistance. This variable was analysed based on previous studies [31], i.e. a KDT_50_ Ratio >2 fold compared with the Kisumu reference susceptible mosquito strain indicates a significant increase of knock-down times.

### Mosquito species identification and kdr L1014 genotyping

Total DNA of single mosquitoes used as controls and those surviving through susceptibility tests was extracted as described by Collins et al. [32]. Each mosquito was identified down to species level using Polymerase Chain Reaction-Restriction Fragment Length Polymorphism (PCR-RFLP) [33]. Alleles at the *kdr* 1014 locus were genotyped in surviving mosquitoes using Hot Oligonucleotide Ligation Assay (HOLA) as described by Lynd et al. [34]. Positive and negative controls were included within each of these analyses to ensure they were performing correctly.

### Data analysis

The 50 % and 95 % knockdown times (KDT_50_ and KDT_95_) for mosquitoes exposed to insecticides were estimated using a log-time probit model [35]. The log-probit analyses were performed using the WIN DL (version 2.0, 1999) software. The KDT_50_ recorded from field-collected mosquitoes were compared with that of the Kisumu reference susceptible strain of *An. gambiae s.s.* by estimates of KDT_50_ Ratios (KDT_50_R). The reversion of knockdown times by PBO was estimated as described by Thomas, Kumar & Pillai [36] using the formula; Reversion of Knockdown time = (1 – (KdT_50 PBO+insecticide_/KdT_50 insecticide_)) x 100. The mortality rates of mosquitoes tested with insecticides alone were compared to that of specimens pre-exposed to PBO by means of a Chi square Mantel Haenszel test. Allelic and genotypic frequencies at the *kdr* 1014 locus were calculated using Genepop Online (Version 4.5.1, [37]).

## Results

### Typology of *Anopheles coluzzii* permanent and semi-permanent larval habitat

*An. coluzzii* larvae were found mainly in natural habitats in fringes of the Wouri River whose flow is generally highly irregular, as well as in ponds, residual puddles, boats, and drains. Details on the most active and permanent and semi-permanent larval breeding sites in Manoka and Youpwe are given in Table 1. The types of these breeding sites were broadly similar across the two study areas, including ponds, boats and drains. Irrespective of the period of survey, the three types of breeding sites were equally represented between the two areas. Furthermore, tire tracks were also very active in Youpwe. They were mostly found in the fringe of the Wouri River (Dahomey, Bord and Nyangadou) as well as in the central camp of the island (Fig. 1).

**Table 1:** *Anopheles coluzzii* most active permanent and semi-permanent larval breeding sites in Manoka and Youpwe

		Geographic coordinates			
Study site	Breeding site N°	Latitude N	Longitude E	Altitude (m)	Type of breeding site
Manoka	MB_1_	03°51'557”	09°37'430”	5	boat
	MB_2_	03°51'514”	09°37'496”	5	pond
	MB_3_	03°51'307”	09°37'223”	4	pond
	MB_4_	03°51'300”	09°37'223”	4	boat
	MB_5_	03°51'421”	09°37'207”	5	pond
	MB_6_	03°51'780”	09°37'410”	7	pond
	MB_7_	03°51'052”	09°37'204”	7	boat
	MB_8_	03°51'042”	09°37'179”	7	drain
Youpwe	YB_1_	04°00'363”	09°41'575”	7	drain
	YB_2_	04°00'248”	09°42'096”	7	drain
	YB_3_	04°00'401”	09°42'040”	7	pond
	YB_4_	04°00'502”	09°41'556”	7	pond
	YB_5_	04°00'427”	09°42'552”	7	boat
	YB_6_	04°00'267”	09°41'060”	7	tire tracks
	YB_7_	04°00'290”	09°41'558”	7	boat
	YB_8_	04°00'545”	09°42'042”	7	tire tracks

*MBx* Manoka breeding site number, *YBx* Youpwe breeding site number

### Status of DDT and pyrethroid resistance in *An. coluzzii* populations

A total of 14 susceptibility tests were carried-out on 784 female *An. gambiae s.l.* from Manoka and 613 from Youpwe and 6 tests with 600 *An. gambiae s.s.* from the Kisumu susceptible reference strain. Twelve tests were conducted with only insecticides and 8 with insecticides plus 4 % PBO synergist. Knockdown and mortality rates in control mosquitoes exposed to silicon treated papers were 0–3 %. The knockdown times for 50 % (KDT_50_) and 95 % (KDT_95_) mosquitoes tested with insecticides alone or insecticides combined with PBO are given in Table 2.

**Table 2:** Knockdown times of the Kisumu *Anopheles gambiae s.s.* and wild *Anopheles coluzzii* strains to insecticides

Period	Strain	Insecticide	N	KDT_50_ (min) [CI_95_]	KDT_95_ (min) [CI_95_]	KDT_50_R
December 2013	Kisumu	0.05% Deltamethrin	100	9.5 [8.4-10.8]	17.3 [15.7-19.4]	-
		0.75% Permethrin	100	8.8 [7.3-11.1]	14.0 [12.6-17.8]	-
		4% DDT	100	19.1 [17.8-21.1]	31.2 [28.5-33.1]	-
	Manoka	0.05% Deltamethrin	114	26.6 [25.1-28.1]	54.5 [50.2-60.2]	2.8
		0.75% Permethrin	100	>60	>60	ND
		4% DDT	105	>60	>60	ND
	Youpwe	0.05% Deltamethrin	130	38.1 [36.4-39.8]	>60	4.0
		0.75% Permethrin	103	>60	>60	ND
		4% DDT	100	>60	>60	ND
April 2014	Manoka	0.05% Deltamethrin	76	40.3 [38.1-43.0]	>60	4.2
		4% DDT	89	>60	>60	ND
	Youpwe	0.05% Deltamethrin	71	38.7 [36.7-40.8]	>60	4.0
April 2014	Kisumu	0.05% Deltamethrin + 4% PBO	100	9.2 [7.8-10.8]	18.4 [17.2-20.4]	-
		0.75% Permethrin + 4% PBO	100	8.8 [7.3-11.1]	17.2 [16.1-20.6]	-
		4% DDT + 4% PBO	100	18.1 [16.9-20.8]	31.0 [28.2-33.4]	-
	Manoka	0.05% Deltamethrin + 4% PBO	100	25.8 [20.9-30.1]	54.9 [44.9-78.8]	2.8
		0.75% Permethrin + 4% PBO	100	57.0 [54.8-60.0]	>60	6.4
		4% DDT + 4% PBO	100	>60	>60	ND
	Youpwè	0.05% Deltamethrin + 4% PBO	109	22.3 [18.7-26.4]	36.9 [30.26-56.4]	2.4
		4% DDT + 4% PBO	100	>60	>60	ND

*PBO* piperonyl butoxide, *N* sample size, *KDT*_50_ and *KDT*_95_ knockdown times for 50% and 95% of the tested samples, *KDT*_*50*_ *R* ratio KDT_50_ wild mosquito sample/ KDT_50_ Kisumu strain; min: minute, *ND* not determined

According to WHO criteria, the Kisumu strain was fully susceptible to the three insecticides with 18–19 min KDT_50_ to DDT, 9–11 min KDT_50_ to permethrin and deltamethrin with or without PBO. The KDT_95s_ were less than 32 min and the mortality rates were 100 % for all insecticides used.

Conversely, the wild mosquito samples from Manoka and Youpwe displayed resistance to DDT, permethrin and deltamethrin. The resistance was expressed by a significant decrease in mortality rates and a matching increase in knockdown times compared with the Kisumu susceptible reference strain, suggesting a kdr-based resistance pattern. DDT and deltamethrin resistance was recorded in December 2013 and in April 2014 as well. Permethrin tests were not performed in April 2014 because there were insufficient mosquito samples due to breeding site flushing. The KDT_50_ and KDT_95_ to deltamethrin were 26–40 min and >54 min respectively, while the knockdown rates did not reach 95 % for DDT and permethrin. Deltamethin KDT_50_ Ratios in Manoka and Youpwe compared with the Kisumu strain ranged from 2.8 to 4.2 fold (Table 2).

**Fig. 3:**
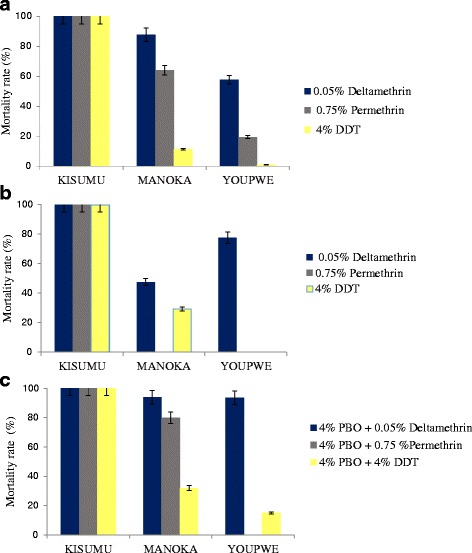
Mortality rates of *Anopheles coluzzii* from Manoka and Youpwe 24 h post-exposure to 4% DDT, 0.75% permethrin or 0.05% deltamethrin. Del: deltamethrin; Perm: permethrin; DDT: Dichlorodiphenyltrichlorethane; PBO: Piperinyl Butoxide; **a**: Mortality rates recorded in December 2013 to insecticides alone; **b**: Mortality rates recorded in April 2014 to insecticides alone; **c**: Mortality rates recorded in April 2014 to insecticides + PBO; Bar absent: insecticide not tested

Mortality rates due to DDT alone or in combination with PBO were mostly lower than 40 %, regardless of the mosquito collection site (Fig. 3). Variable mortality rates were however recorded with deltamethrin and permethrin, depending on the period and sites of mosquito collection. In Manoka, the level of deltamethrin resistance increased from December 2013 (88 % mortality) to April 2014 (48 % mortality) (X^2^ = 21.68, df = 1, *p* < 0.001). By contrast in Youpwe, deltamethrin resistance decreased from December 2013 (58 % mortality) to April 2014 (77 % mortality), although the difference was not significant (X^2^ = 2.05, df = 1, *p* > 0.1). Pre-exposure of mosquitoes to PBO resulted in 36-42 % reversion of knockdown times. Furthermore, the mortality rates significantly increased (7.56 ≤ X^2^ ≤ 48.63, df = 1, *p* < 0.01) as a result of this pre-exposure (Fig. 3).

### Mosquito species, *kdr* L1014 allelic and genotypic frequencies

**Fig. 4:**
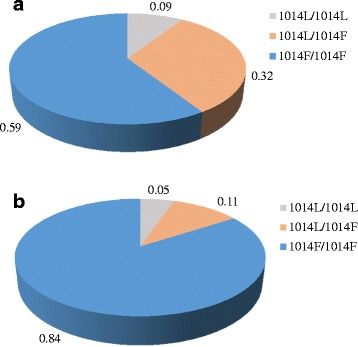
*Kdr* 1014 genotypic frequencies within mosquitoes surviving exposure to insecticides in December 2013. **a**: *kdr* 1014 genotypic profile in Manoka (sample size: 44); **b**: *kdr* 1014 genotypic profile in Youpwe (sample size: 76)

A subsample of 400 *An. gambiae s.l.* (204 from Manoka and 196 from Youpwe) analyzed using the PCR-RFLP method were all found to belong to the *An. coluzzii* species. Among these specimens, 280 (160 from Manoka and 120 from Youpwe) represent 44 % of the 630 specimens previously used as control during susceptibility tests, while 120 specimens (44 from Manoka and 76 from Youpwe) were randomly selected among the 403 survivors to DDT, permethrin or deltamethrin (≈30 %). Among the 120 survivors that were submitted to *kdr* 1014 genotyping, the *kdr* 1014 F allele was found at 88 % and 75 % allelic frequencies in survivors from Youpwe and Manoka, respectively (Table 3). None of the analyzed specimens carried the *kdr* 1014S allele. Three kdr genotypes were recorded: 1014 L/1014L homozygote susceptible, 1014 L/1014F heterozygote, 1014 F/1014F homozygote resistant (Fig. 4). The predominant genotype was 1014 F/1014F (60 % and 84 % in Manoka and Youpwe samples respectively), followed by 1014 L/1014F (32 % and 11 % in Manoka and Youpwe samples respectively). The homozygotes 1014 L/1014L were also present although at very low frequencies less than 10 %.

**Table 3:** *Kdr* L1014 allelic frequencies in survivor *An. coluzzii* from Manoka and Youpwe, December 2013

Strain	N	*Kdr* 1014 allelic frequency (%) [CI_95_]		
		1014L	1014F	1014S
Manoka	44	25 [16.4-35.4]	75 [64.63-82.6]	0
Youpwe	76	12 [7.1-17.6]	88 [82.3-93.1]	0

*N* Sample size, *CI95* confidence interval at 95%, *1014L* 1014 Leucine, *1014F* 1014 Phenylalanine, *1014S* 1014 Serine

## Discussion

Irrespective of the period of survey, three types of permanent or semi-permanent breeding sites for *An. coluzzii* were commonly recorded among the most active larval habitats in the Manoka rural island area and the Youpwe suburban area. These were drains, ponds and boats. More interestingly, at least a quarter of the active water bodies in both localities were boat-breeding sites, highlighting a previously unreported important niche for *An. coluzzii* breeding in these areas.

Although Tene-Fossog and colleagues [38] predicted a predominance of *An. coluzzii* along the coastline of west and central Africa, there was still limited information on *An. coluzzii* occurrence and larval habitat on the Manoka island area. Small, temporary sunny water bodies, relatively clean and mostly without overhanging vegetation have been recognized by several studies as preferred breeding habitats for *An. gambiae s.l.* [39]. Other studies have reported the breeding of *An. gambiae s.l.* in large permanent and polluted water bodies [40, 41]. More precisely, ecological niches of species of the *An. gambiae* complex were found to segregate according to habitat type and temporality [42]. In Cape Coast (Ghana), *An. coluzzii* was reported to actively breed in diverse habitats, including footprints, tyre tracks, rain pools during the rainy season, and choke gutters and other organic polluted habitats during the dry season [43]. Although temporary breeding sites may be equally important in the ecology of *An. coluzzii*, they were not very active during the survey periods in Manoka and Youpwe. We therefore focused the current study on permanent and semi-permanent breeding sites which were positive during the two larvae collection surveys. The positivity of permanent and semi-permanent breeding sites for *An. coluzzi* in the current study was consistent with Gimonneau et al. [12], who reported that “permanent-anthropic” habitats are mostly exploited by *An. coluzzii* (former M form), while “temporary natural” habitats are colonised by *An. gambiae s.s* (former S form) and *An. arabiensis*. Furthermore, the exclusive identification of *An. coluzzii* in the current study supports previous reports of this mosquito species representing the dominant malaria vector species in the Littoral Region of Cameroon [11].

On the other hand, interbreeding between mosquito populations may influence their dispersal and the subsequent evolution of insecticide resistance. The present study revealed insecticide resistance in two interbreeding *An. coluzzii* vector populations, and highlighted seasonal variations in resistance patterns (wet/dry). Similar variations in susceptibility of *An. gambiae s.l.* populations to insecticides were reported in some African countries [44–47] and North Cameroon as well [48]. They were linked to seasonal immigration of *kdr*-resistant *An. gambiae s.s.* mosquitoes originating from the neighbouring cotton fields, temporal variations in species composition within the *An. gambiae s.l.* complex, or cycles of insecticide treatments for agriculture and/or public health purposes. In Manoka and Youpwe, seasonal variations of resistance levels over the year might result from local fluctuations of vector population dynamics according to rainy events and human activities related to agriculture and household protection against mosquito bites (e.g. use of coils, mats, etc. purchased from the market). In addition, these variations may be related to the migration of *An. coluzzii* specimens through boat transportation between the two areas, and to a lesser extent to changes in species composition, since *An. gambiae s.s.* has been reported in some quarters in Douala.

Furthermore, the rapid scale-up of insecticide-based malaria control measures in Africa during the first decade of the 2000s has had measurable effects on malaria vectors, especially on *An. gambiae* and *An. coluzzii,* which are highly dependent on humans for blood and indoor resting sites. In some cases, selection pressure from these measures has been strong enough to drive *An. gambiae* and *An. coluzzii* to local extinction [49]. In other cases, the selection pressure has resulted in rapid evolution of pyrethroid resistance and increase of *kdr* L1014F allelic frequencies, as reported in Douala and Yaounde in Cameroon [50]. The two subsequent nationwide LLINs distribution campaigns launched in 2011 and 2015, in addition to the use of insecticides in agriculture would be expected to impact on local malaria vector populations.

Mosquito samples used in the current study were collected during two periods (December 2013 and April 2014) in order to reflect seasonal variations of DDT, permethrin and deltamethrin resistance in *An. gambiae s.l.* from Manoka and Youpwe. However, the low productivity and the flush of larval breeding sites during the dry and rainy seasons respectively precluded us to complete all the susceptibility tests for the three targeted insecticides, with or without PBO. Nevertheless, the 14 tests that were successfully performed provided an overview of DDT, permethrin and deltamethrin resistance phenotypes and the *kdr* 1014 genotypes in *An. coluzzii* populations from the study sites. The level of *An. coluzzii* resistance to DDT and pyrethroids as well as the *kdr* 1014 F allelic frequencies recorded in the current study are higher than those previously reported in *An. gambiae s.s* and *An. coluzzii* from some remote districts of the Douala city [51]. These results testify that the spread of the *kdr* alleles is an ongoing process in *An. gambiae s.l.* mosquito populations from Cameroon [18, 51, 52], as well as elsewhere in Central Africa [53, 54]. The high frequencies of *kdr* L1014F allele in surviving samples (75-88 %), with predominance of the *kdr* 1014 F/1014F homozygote genotype (60-84 %), suggests a strong involvement of this allele in the resistance phenotype. The *kdr* 1014 F allele being recessive, the presence of 1014 L/1014F heterozygotes (11–32 %), as well as a few 1014 L/1014L homozygotes (<10 %) in surviving samples suggest involvement of other resistance mechanisms. Indeed, pre-exposure of mosquitoes to PBO resulted in reversion of pyrethroid resistance in both tested populations, suggesting involvement of P450 oxidases in this resistance [55]. The synergistic effect of PBO and high frequencies of *kdr* L1014F allele in surviving specimens strongly suggests multiple pyrethroid resistance mechanisms in *An. coluzzii* from Manoka and Youpwe. These findings fit well in the overall picture presented in previous surveys in Douala and other settings in Cameroon [19].

In West Africa, *An. coluzzii* seems to have responded to insecticide selection pressure by co-opting pyrethroid and DDT resistance in the form of the L1014F mutation from *An. gambiae* through adaptive introgression [56]. The selection of the *kdr* 1014 F allele is likely stronger in *An. coluzzii* than in sympatric *An. gambiae*, owing to either (i) the need for the resistance allele in the presence of insecticide to overcome the selective disadvantage of *An. gambiae* or (ii) phenotypic penetrance of L1014F differing in the *An. gambiae* genetic background [57]. In the Bioko Island (Equatorial Guinea, neighbor to South Cameroon), a high and unusual distribution of *kdr* L1014F frequencies in *An. coluzzii* (former M molecular form of *An. gambiae s.s.*) was also reported [58]. In some quarters in Douala, both *kdr* L1014S and L1014F alleles have been reported in *An. coluzzii* specimens surviving susceptibility tests, at frequencies of 4 % and 38 % respectively [19]. The absence of the L1014S allele in samples from Manoka and Youpwe suggests its selective disadvantage compared with the L1014F allele in these areas. The L1014F allele may have arisen in coastal Cameroon from in situ mutation events, and evolved through gene flow or local selection pressure due to insecticide use in vector control interventions and individual protection tools against mosquito bites [59]. Indeed, DDT was massively used for indoor residual spraying in the framework of the pilot malaria eradication programme in Douala during the 1950s [60]. Furthermore, Desfontaines et al. [61] reported the widespread use of insecticides for household protection against mosquito bites in Douala.

The influence of evolutionary forces (mutations, gene flow and selection) in shaping *An. coluzzii* populations from Youpwe and Manoka might therefore be counterbalanced by ecological fitness costs, resulting in the development of insecticide resistance.

## Conclusion

The similarity of *An. coluzzii* distribution in the Manoka island area and Youpwe mainland quarter, its presence in the same range of breeding sites, and regular connection between the two settings by boat transportation and patterns of insecticide resistance suggest gene flow between mosquito populations from the two areas. These findings reemphasize the spread of insecticide resistance in species of the *An. gambiae* complex from various ecological areas in Cameroon, and the need for adequate resistance management strategies. The level of resistance may increase during the coming years following the second nationwide LLIN distribution by the National Malaria Control Programme. New and innovative vector control tools are therefore needed to complement LLINs in this country. Since permanent larval habitats have been identified in the study sites, larval source management strategies may be trialed in these settings as a potential complementary vector control strategy, especially in fishing camps where the risk of outdoor malaria transmission is high.

### Ethics and consent to participate

According to the guidelines of the “Comité d’Ethique de la Recherche et de la Santé en Afrique Centrale (CERSAC)” hosted by OCEAC, no formal ethics approval, nor informed consent was required in this particular study, since human populations were not involved as participants of the study. Furthermore, mosquito larvae were collected form water bodies outdoors.

### Consent to publish

Not applicable.

### Availability of data and materials

All the data supporting our findings is contained within the manuscript.

## Abbreviations

*An*: *Anopheles*
DDT: dichloro-diphenyl-trichlorethane
DNA: deoxyribonucleic acid
HOLA: hot oligonucleotide ligation assay
ISRT-Health: International Society for Health Research and Training
*Kdr*: knockdown resistance
LLINs: long-lasting insecticidal nets
L1014F: leucine to phenylalanine mutation at 1014 locus
L1014S: leucine to serine mutation at 1014 locus
OCEAC: organisation de coordination pour la lutte contre les Endémies en Afrique Centrale
OMS: Organisation Mondiale de la Santé
PBO: piperonyl butoxide
PCR-RFLP: polymerase chain reaction-restriction fragment length polymorphism
KDT_50_: time of knockdown for 50 % tested specimens
KDT_50_R: ratio times of knockdown for 50 % between tested population and the Kisumu susceptible strain
KDT_95_: time of knockdown for 95 % tested specimens
WHO: World Health Organization

## References

[1] Organisation Mondiale de la Santé (OMS). Stratégie technique mondiale de lutte contre le paludisme 2016–2030. Genève, Suisse: Organisation mMondiale de la Santé; 2015.

[2] Service MW (1991). Agricultural development and arthropod-borne diseases: a review. Revu Saude Publica.

[3] World Health Organization (WHO). Larval source management: A supplementary measure for malaria vector control (operational manual). Geneva, Switzerland: World Health Organization; 2013.

[4] Programme National de lutte Contre le Paludisme (PNLP). Rapport d’activités 2012 du Programme National de Lutte contre le Paludisme. Yaoundé, Cameroun: Ministère de la Santé; 2013.

[5] Same-Ekobo A (2005). Aspects épidémiologiques du paludisme au Cameroun. J Cam Acad Sci.

[6] Mouchet J, Carnevale P, Coosemans M, Julvez J, Manguin S, Lenoble RD, Sircoulon J. Biodivesité du paludisme dans le monde. Editions John Libbey Eurotext Paris. 2004

[7] Antonio-Nkondjio C, Kerah CH, Simard F, Awono-Ambene P, Chouaїbou M, Tchuinkam T, et al. Complexity of the malaria vectorial system in Cameroon: contribution of secondary vectors to malaria transmission. J Med Entomol. 2006;43:1215–21. 10.1603/0022-2585(2006)43[1215:cotmvs]2.0.co;2 17162956

[8] Wondji C, Simard F, Petrarca V, Etang J, Santolamazza F, DellaTorre A, et al. Species and populations of the *Anopheles gambiae* Complex in Cameroon with special Emphasis on Chromosomal and Molecular Forms of *Anopheles gambiae* s.s. J Med Entomol. 2005;42:998–1005. 10.1093/jmedent/42.6.998 16465741

[9] Lehmann T, Diabaté A (2008). The molecular forms of *Anopheles gambiae*: A phenotypic perspective. Infect Genet Evol.

[10] Costantini C, Ayala D, Guelbeogo W, Pombi M, Some C, Bassole I (2009). Living at the edge: biogeographic patterns of habitat segregation conform to speciation by niche expansion in *Anopheles gambiae*. BMC Ecol.

[11] Simard F, Ayala D, Kamdem G, Pombi M, Etouna J, Ose K (2009). Ecological niche partitioning between *Anopheles gambiae* molecular forms in Cameroon: the ecological side of speciation. BMC Ecol.

[12] Gimonneau G, Pombi M, Choisy M, Morand S, Dabiré RK, Simard F (2012). Larval habitat segregation between the molecular forms of the mosquito, *Anopheles gambiae* in a rice field area of Burkina Faso, West Africa. Med Vet Entomol.

[13] Programme National de Lutte contre le paludisme (PNLP). Plan stratégique national de lutte contre le paludisme 2011–2015. Yaoundé, Cameroun: Ministère de la Santé; 2011.

[14] Etang J, Manga L, Chandre F, Guillet P, Fondjo E, Mimpfoundi R, et al. Insecticide susceptibility status of *Anopheles gambiae* s.l. (Diptera: Culicidae) in the Republic of Cameroon. J Med Entomol. 2003;40:491–7. 10.1603/0022-2585-40.4.491 14680116

[15] Chouaїbou M, Etang J, Brevault T, Nwane P, Hinzoumbé CK, Mimpfoundi R, et al. Dynamics of insecticide resistance in the malaria vector *Anopheles gambiae* s.l. from an area of extensive cotton cultivation in Northern Cameroon. Trop Med Int Hlth. 2008;13:1–11. 10.1111/j.1365-3156.2008.02025.x 18248566

[16] Antonio-Nkondjio C, TeneFossog B, Ndo C, MenzeDjantio B, ZebazeTogouet S, Awono-Ambene P, et al. *Anopheles gambiae* distribution and insecticide resistance in the cities of Douala and Yaoundé (Cameroon): influence of urban agriculture and pollution. Mal J. 2011;10:154. 10.1186/1475-2875-10-154 PMC3118161 21651761

[17] Etang J, Manga L, Toto JC, Guillet P, Fondjo E, Chandre F (2007). Spectrum of metabolic-based resistance to DDT and pyrethroids in *Anopheles gambiae s.l* populations from Cameroon. J Vect Eco.

[18] Nwane P, Etang J, Chouaїbou M, Toto JC, Mimpfoundi R, Simard F (2011). Kdr-based insecticide resistance in *Anopheles gambiae* s.s populations in Cameroon: spread of the L1014F and L1014S mutations. BMC Res notes.

[19] Nwane P, Etang J, Chouaїbou M, Toto JC, Koffi A, Mimpfoundi R, Simard F (2013). Multiple insecticide resistance mechanisms in *Anopheles gambiae* s.l. populations from Cameroon, Central Africa. Parasites and Vectors.

[20] Maps and satellite images. http://en.geo-trotter.com/search.htm. Retrieved; 2013.

[21] WMO (World Meteorological Organization). World Weather Information Service-Douala. Douala, Cameroon: World Meteorological Organization; 2012. Retrieved.

[22] Weather base. Historical Weather for Douala, Cameroun. Retrieved; 2012.

[23] Olivry JC. Fleuves et Rivières du Cameroun. Bondy : Collection «Monographies hydroliques ORSTOM »;1986.

[24] Moutila BL. Pression et dynamique de l'espace côtier à mangrove de Youpwe (Douala-Cameroun). Dissertation, Master of Geography. The University of Douala. 2011;111p.

[25] https://en.wikipedia.org/wiki/Manoka. Accessed 14 Oct 2013.

[26] Stein M, Ludueña-Almeida F, Willener JA, Almirón WR (2011). Classification of immature mosquito species according to characteristics of the larval habitat in the subtropical province of Chaco, Argentina. Mem Inst Oswaldo Cruz, Rio de Janeiro.

[27] Service MW. Mosquito ecology, field sampling methods vector biology and control. 2nd ed. Liverpool School of Tropical Medicine; 1993.

[28] Gillies MT, Coetzee M (1987). A supplement to the Anophelinae of Africa south of the Sahara (Afrotropical region).

[29] Gillies MT, de Meillon B (1968). The Anophelinae of Africa South of the Sahara (Ethiopian zoogeographical region).

[30] WHO (World Health Organization). Test procedures for insecticide resistance monitoring in malaria vectors. Geneva, Switzerland: World Health Organization; 2013.

[31] Chandre F, Frederic D, Sylvie M, Cecile B, Carnevale P (1999). P. Guillet. Pyrethroid cross resistance spectrum among populations of *Anopheles gambiae* s.s. from Côte d’Ivoire. J Am Control Assoc Inc.

[32] Collins FH, Mendez MA, Razmussen MO, Mehaffey PC, Besansky NJ, Finnerty VA (1987). Ribosomal RNA gene probe differentiates member species of *Anopheles gambiae* complex. Am J Trop Med Hyg.

[33] Fanello C, Santolamazza F, Della TA (2002). A Simultaneous identification of species and molecular forms of the *Anopheles gambiae* complex by PCR-RFLP. Med Vet Entomol.

[34] Lynd A, Ranson H, McCall PJ, Randle NP, Black IV WC, Walker ED, et al. A simplified high-throughput method for pyrethroid knockdown resistance (kdr) detection in *Anopheles gambiae*. Mal J. 2005;4:16. 10.1186/1475-2875-4-16 PMC555548 15766386

[35] Finney DJ (1971). Probit analysis.

[36] Thomas A, Kumar S, Pillai MKK (1991). Piperonyl butoxide as a counter measure for deltamethrin resistance in *Culex quinquefasciatus* Say. Entomol.

[37] Rousset F (2008). genepop’007: a complete re-implementation of the genepop software for Windows and Linux. Mol Ecol Res.

[38] Tene Fossog B, Ayala D, Acevedo P, Kengne P, Abeso Mebuy INA (2015). Habitat segregation and ecological character displacement in cryptic African malaria mosquitoes. Evol Appl.

[39] Muirhead-Thomson RC (1951). Mosquito behaviour in relation to malaria transmission and control in the tropics.

[40] Sattler MA, Mtasiwa D, Kiama M, Premji Z, Tanner M, Killeen GF (2005). Habitat Characterization and spatial distribution of Anopheles sp. Mosquito larvae in Dares Salaam (Tanzania) during an extended dry period. Malar J.

[41] Kamdem C, Tene Fossog B, Simard F, Etouna J, Ndo C, Kengne P (2012). Anthropogenic habitat disturbance and ecological divergence between incipient species of the malaria mosquito *Anopheles gambiae*. PLoS One.

[42] Edillo FE, Toure YT, Lanzaro GC, Dolo G, Taylor CE (2002). Spatial and habitat distribution of *Anopheles gambiae* and *Anopheles arabiensis* (Diptera : Culicidae) in Banambani Village. Mali J Med Entomol.

[43] Kudom AA (2015). Larval ecology of *Anopheles coluzzii* in Cape Coast, Ghana: water quality, nature of habitat and implication for larval control. Malar J.

[44] Diabate A, Baldet T, Chandre F, Akogbeto M, Guiguemde TR, Darriet F, et al. The role of agricultural use of insecticides in resistance to pyrethroids in *Anopheles* gambiae *s.l.* in Burkina Faso. Am J Trop Med Hyg. 2002;67:617–22. 10.4269/ajtmh.2002.67.617 12518852

[45] Djegbe I, Boussari O, Sidick A, Martin T, Ranson H, Chandre F, et al. Dynamics of insecticide resistance in malaria vectors in Benin: first evidence of the presence of L1014S kdr mutation in *Anopheles gambiae* from West Africa. Malar J. 2011;10:261. doi:10.1186/1475-2875-10-261. 10.1186/1475-2875-10-261 PMC3179749 21910856

[46] Abdalla H, Wilding CS, Nardini L, Pignatelli P, Koekemoer LL, Ranson H, et al. Insecticide resistance in *Anopheles arabiensis* in Sudan: temporal trends and underlying mechanisms. Parasites Vectors. 2014;7:213. doi:10.1186/1756-3305-7-213. 10.1186/1756-3305-7-213 PMC4026821 24886129

[47] Ranson H, Abdalla H, Badolo A, Guelbeogo WM, Kerah-Hinzoumbe C, Yangalbe-Kalnone E, et al. Insecticide resistance in *Anopheles gambiae*: data from the first year of a multi-country study highlight the extent of the problem. Malar J. 2009;8:299. doi:10.1186/1475-2875-8-299. 10.1186/1475-2875-8-299 PMC2804687 20015411

[48] Chouaïbou M, Etang J, Brévault T, Nwane P, Kérah HC, Mimpfoundi R, et al. The dynamics of insecticide resistance in the malaria vector *Anopheles gambiae s.l.* from an area of extensive cotton cultivation in Northern Cameroon. Trop Med Int Hlth. 2008;13:476–86. 10.1111/j.1365-3156.2008.02025.x 18248566

[49] Govella NJ, Chaki PP, Killeen GF (2013). Entomological surveillance of behavioural resilience and resistance in residual malaria vector populations. Mal J.

[50] Antonio-Nkondjio C, Tene Fossog B, Kopya E, Poumachu Y, Menze Djantio B, Ndo C, et al. Rapid evolution of pyrethroid resistance prevalence in *Anopheles gambiae* populations from the cities of Douala and Yaoundé (Cameroon). Mal J. 2015;14:155. 10.1186/s12936-015-0675-6 PMC4403825 25879950

[51] Nwane P, Etang J, Chouaibou M, Toto JC, Kerah-Hinzoumbé C, Mimpfoundi R, et al. Trends in DDT and pyrethroid resistance in *Anopheles gambiae* s.s. Populations from urban and agro-industrial settings in southern Cameroon. BMC Infect Dis. 2009;9:163. 10.1186/1471-2334-9-163 PMC2764715 19793389

[52] Etang J, Fondjo E, Chandre F, Morlais I, Brengues C, Nwane P, Chouaïbou M, Djemaï A, Simard F (2006). First report of the kdr mutations in the malaria vector *Anopheles gambiae* from Cameroon. Am J Trop Med Hyg.

[53] Pinto J, Lynd A, Elissa N, Donnelly MJ, Costa C, Gentile G, Caccone A, Do Rosário VE (2006). Co-occurrence of East and West African *kdr* mutations suggests high levels of resistance to pyrethroid insecticides in *Anopheles gambiae* from Libreville, Gabon. Med Vet Entomol.

[54] Santolamazza F, Calzetta M, Etang J, Barrese E, Dia I, Caccone A, Donnelly MJ, Petrarca V, Simard F, Pinto J, della Torre A (2008). Distribution of knockdown resistance mutations in *Anopheles gambiae* molecular Forms in West and West-Central Africa. Malar J.

[55] Chouaïbou M, Zivanovic GB, Knox TB, Pates HJ, Bonfoh B (2013). Synergist bioassays: A simple method for initial metabolic resistance investigation of field *Anopheles gambiae* s.l. populations. Acta Trop.

[56] Weill M, Chandre F, Brengues C, Manguin S, Akogbeto M, Pasteur N, Guillet P, Raymond M (2000). The Kdr mutation occurs in the Mopti form of *Anopheles gambiae s.s.* through introgression. Inst Mol Biol.

[57] Norris LC, Main BJ, Lee Y, Collier TC, Fofana A, Lanzaro GC, Cornel AJ (2015). Adaptive introgression in an African malaria mosquito coincident with the increased usage of insecticide-treated bed nets. Proc Natl Acad Sci U S A.

[58] Reimer LJ, Tripet F, Slotman M, Spielman A, Fondjo E, Lanzaro GC (2005). An unusual distribution of the *kdr* gene among populations of *Anopheles gambiae* on the Island of Bioko, Equatorial Guinea. Insect Mol Biol.

[59] Etang J, Vicente JL, Nwane P, Chouaibou M, Morlais I, Do Rosario VE, Simard F, Awono-Ambene HP, Toto JC, Pinto J (2009). Polymorphism of intron-1 in the voltage-gated sodium channel of *Anopheles gambiae* s.s. populations from Cameroon with emphasis on insecticide knockdown resistance mutations. Mol Ecol.

[60] Livadas G, Mouchet J, Gariou J, Chastang R (1958). Peut-on envisager l’éradication du paludisme dans la région forestière du Sud-Cameroun ?. Riv Malariol.

[61] Desfontaines M, Gelas H, Cabon H, Goghomu A, Kouka-Bemba D, Carnevale P (1990). Evaluation des pratiques et des coûts de lutte antivectorielle à l'échelon familial en Afrique centrale. II-Ville de Douala (Cameroun), juillet 1988. Ann Soc Belg Med Trop.

